# Observational vignette study to examine patient and healthcare provider perceived impact of asthma-related exacerbations in the US

**DOI:** 10.1186/s40248-019-0196-1

**Published:** 2019-11-05

**Authors:** Phaedra T. Johnson, Christopher F. Bell, John White, Breanna Essoi, Linda Nelsen, Carlyne M. Averell

**Affiliations:** 1 Health Economics and Outcomes Research, Optum, 11000 Optum Circle, Eden Prairie, MN 55344 USA; 2 US Value, Evidence, and Outcomes, GlaxoSmithKline, 5 Moore Drive, Reesearch Triangle Park, NC 27709-3398 USA; 3 Value, Evidence, and Outcomes, GlaxoSmithKline, 1250 S Collegeville Road, Collegeville, PA 19426 USA

**Keywords:** Asthma, Exacerbations, Burden, Vignette, Patient, Healthcare provider

## Abstract

**Background:**

Little is known about how patients and healthcare providers (HCPs) perceive the impact of asthma-related exacerbations. This study examined the impact of asthma-related exacerbations on patients’ lives from these different perspectives.

**Methods:**

Web-based surveys were administered to a US sample of adult patients with asthma, and HCPs. Participants reviewed six vignettes describing two hypothetical patients with asthma (25-year-old/single/unemployed/no dependents; and 45-year-old/married/employed/two young children) experiencing mild, moderate, or severe exacerbations and rated the impact on eight measures: EuroQoL-5 Dimensions (mobility, self-care, usual activities, pain/discomfort, and anxiety/depression), sleep, household costs, and medical costs. The proportions reporting impact for each measure were calculated for each vignette; and patient responses were compared with HCP responses.

**Results:**

302 patients with asthma and 300 HCPs completed the survey. As exacerbation severity increased, a higher proportion of patients and HCPs reported impact of exacerbations on patients with asthma. Compared with HCPs, a greater proportion of patients reported problems with pain/discomfort related to mild and moderate exacerbations. Compared with patients, HCPs were more likely to indicate sleep impact, mobility problems, and financial burden across all exacerbation severity levels; self-care problems with moderate and severe exacerbations; and problems with usual activities and anxiety/depression for severe exacerbations.

**Conclusions:**

Understanding the distinctions between how patients and HCPs perceive the impact of exacerbations is important for optimizing patient care. HCPs may be less aware of patient’s concerns about exacerbation-related pain/discomfort. Studies are needed to further understand patient-HCP interactions regarding asthma-related exacerbations.

**Electronic supplementary material:**

The online version of this article (10.1186/s40248-019-0196-1) contains supplementary material, which is available to authorized users.

## Background

Asthma is a common chronic inflammatory disorder of the airways, affecting over 8% of the population of the United States (US) [1]. The primary goals of asthma treatment are to achieve the best possible clinical control and reduce the future risk of adverse outcomes [2, 3]. The American Thoracic Society/European Respiratory Society (ATS/ERS) Task force defines asthma control as ‘the extent to which the manifestations of asthma have been reduced or removed by treatment’ [2]. Assessment of asthma control should incorporate both current clinical control (including symptoms and the extent that patients can continue with daily activities and achieve optimum health-related quality of life [HRQoL]) and future risk (including exacerbations and accelerated decline in lung function) [2, 4].

Poorly controlled asthma is associated with reduced HRQoL and increased healthcare resource utilization and costs compared with well-controlled asthma [5–9]. Prevention of asthma-related exacerbations is an important component of establishing asthma control. In clinical practice, asthma-related exacerbations are recognized as ‘episodes that are troublesome to patients and prompt a need for a change in treatment’ and are identified clinically as being outside the normal range of day-to-day variation for an individual patient [2]. Exacerbations are generally classified as mild, moderate, or severe based on the patient’s symptoms and other clinical factors, such as requirements for change in treatment or hospital admissions [2]. Definitions of asthma-related exacerbations have been put forward by the ATS/ERS Task Force, however, there are currently no established consensus guidelines to specifically define the severity of asthma-related exacerbations in clinical practice or for the optimal management of exacerbations according to severity [2]. The lack of clinical guidelines, which are recognized as a core component of optimal patient-centered care, presents a challenge for healthcare providers (HCPs) as it may limit their ability to treat their patients optimally and consistently. Further complicating the clinical challenges, HCPs and patients may not share an understanding of what constitutes a ‘mild’, ‘moderate’, or ‘severe’ exacerbation, nor how exacerbations impact patients’ lives. For example, patients may not inform their HCPs of the full exacerbation-related impact that they experience, including the economic burden associated with asthma, which continues to be substantial in terms of both healthcare costs and work loss [10].

This study used a vignette-based research design to examine the perceived impact of asthma-related exacerbations on patients with asthma, from the perspective of both the patients themselves and from HCPs, and the relationship between these perceptions.

## Methods

### Study design

This non-interventional study utilized internet-based cross-sectional surveys administered to two independent US cohorts: adult patients with asthma (patient cohort) and HCPs who treat patients with asthma (HCP cohort). Participant recruitment and pilot testing were conducted by Research Now (Dallas, TX, US), a global research service and online panel provider. The study was conducted using a multi-step approach. The first step was development of the initial vignette-based survey instrument, which was guided by online bulletin board focus groups consisting of 14 patients and 11 HCPs who were members of an opt-in online research panel (20/20 Research, Nashville, TN, US). The instrument was then pre-tested in a sample of 6 patients and 7 HCPs, followed by pilot testing of the revised instrument in a cohort of 30 patients and 30 HCPs prior to implementation of the full survey.

### Participant population

Study participants were enrolled via email invitations that contained a link to cohort-specific internet-based surveys, which were conducted between May 25, 2017 and June 2, 2017. Prior to completing the survey, all participants completed cohort-specific screening questions to confirm eligibility. The invitation email included an Institutional Review Board (IRB)-approved informed consent statement, with participants providing consent by completing the survey. Participants in the patient cohort were required to be ≥18 years of age with a self-reported diagnosis of asthma. Participants in the HCP cohort were required to be HCPs who were currently treating patients with asthma, including primary care practitioners, allergists, pulmonologists, and nurses. An additional cohort of adults moderately to very involved in the informal care of patients diagnosed with asthma (caregiver cohort) was included in the development and administration of the survey (results not shown). To ensure the cohorts were representative of asthma distribution in the US population, enrollment targets included ≥60% female participation in the patient cohort and ≥ 60% primary care or family practice participants in the HCP cohort. After the targeted number of 300 surveys per cohort (patient and HCP) was reached, the survey website was closed. Patients received $5.50 in the form of ‘e-rewards’ and HCPs were paid $32.50 for their participation in the study.

### Study measures

#### Vignettes

Vignettes were developed using guidance from previously-published vignette-based analyses [11–15] and described two hypothetical patients: Patient A, “25-year-old college student who is single, not employed, and has no dependents”; and Patient B, “45-year-old, married parent of two young children who also works full time”. Study participants were presented with six vignettes describing the experience of a mild, moderate, or severe exacerbation for the hypothetical patients, in a randomized order (Additional file 1). Exacerbation definitions are provided in Additional file 2. For each vignette, participants rated the impact of the asthma exacerbation on the vignette patient on a scale of 1 (no problems/impact on sleep/financial burden/pain or discomfort, or not anxious or depressed) to 5 (unable to or extreme pain or discomfort/impact on sleep/financial burden, or extremely anxious or depressed) for the following measures: five health domains (mobility, self-care, usual activities, pain/discomfort, and anxiety/depression) derived from the 5-domain, 5-level QoL survey instrument developed by the EuroQol Group (EQ-5D-5 L) [16]; sleep, using a single question derived from the COPD and Asthma Sleep Impact Scale (CASIS) [17]; and economic burden (household and medical costs).

The terms ‘problems’, ‘impact’, and ‘burden’ are all used to describe the various issues that patients with asthma face; however, in this manuscript we have used the term ‘impact’ as a broad term to describe these issues as a whole, except when discussing a specific factor (e.g. economic burden).

#### Patient-reported measures

Patients self-reported their asthma history, comorbid conditions, exacerbation history and current asthma medication use, and completed several patient-reported outcomes (PROs) including the EQ-5D-5 L (including the EQ-Visual Analogue Scale [EQ-VAS]) [16] plus the Asthma Control Test (ACT) [18]. ACT results were categorized as poorly-controlled (score < 16), somewhat-controlled (score 16–19), or well-controlled (score > 19). Patients also reported the impact of asthma on their sleep over the past 4 weeks using the CASIS, and occupational and interpersonal impairment due to asthma using the six-item Work Productivity and Activity Impairment (WPAI) Specific Health Problem instrument [19]. Patients were also asked whether they agreed with various belief statements regarding asthma exacerbations, including: all patients with asthma are at risk of developing an asthma attack; asthma attacks should be categorized by their severity (mild, moderate, and severe); asthma attacks have an impact on their lives; asthma attacks can have a varying impact on their lives, depending on their severity; asthma attacks have an impact on other people in their lives; and they worry about having an asthma attack. Patient demographic and sociodemographic characteristics were also collected.

#### HCP-reported measures

HCP demographics and practice characteristics were collected, including gender, specialty, years in practice, degree, practice setting, number of patients with asthma that were treated in the past year, severity of asthma across all patients treated in the past year, and number of patients treated for exacerbations in the prior month, by severity. HCPs were also asked if they agree with several belief statements regarding asthma exacerbations, including: all patients with asthma are at risk of developing an exacerbation; asthma exacerbations should be categorized by their severity (mild, moderate, and severe); asthma exacerbations have an impact on their patients’ lives; asthma exacerbations can have a varying impact on their patients’ lives, depending on their severity; and they believe their patients worry about having an asthma exacerbation.

### Statistical analysis

All variables were analyzed descriptively using SAS statistical software package version 9.4 (SAS Institute Inc., Cary, NC, US). For each vignette, the proportions of participants reporting impact for the EQ-5D-5 L domains, sleep, and financial burden measures were calculated and compared between patients and HCPs using appropriate tests (e.g., *t*-test, chi-square test) based on the distribution of the measure. A *p* ≤0.05 was considered significant; *p* were not adjusted for multiplicity.

## Results

### Study population

Overall, 491 patients and 449 HCPs accessed their respective survey using the web link. Out of those, 113 patients and 87 HCPs initiated but did not complete the survey, and 76 patients and 62 HCPs did not meet the inclusion criteria and/or the sample size quotas were met; therefore, the final study sample consisted of 302 patients and 300 HCPs. The mean survey completion time was 16 min for patients and 20 min for HCPs.

### Patient characteristics

Patient demographics and clinical characteristics are described in Table 1. Patients had a mean (standard deviation [SD]) age of 48.2 (16.7) years, 61% were female and 77% were white. Over half (52%) had asthma for ≥20 years, the majority were currently prescribed a controller medication (77%) and/or a rescue medication (79%), and most had some form of health insurance coverage (94%). Patient characteristics as measured by PROs completed at study entry are presented in Table 2. Regarding EQ-5D-5 L domains, patients were most likely to report pain/discomfort and anxiety/depression as problematic (54 and 47%, respectively), and 49% reported that their asthma impacted their sleep. The mean (SD) ACT score was 20.3 (4.3) and most patients’ asthma was well-controlled (64%), with 16 and 21% reporting poor or somewhat controlled asthma, respectively. At the time of the survey, 57% of patients were employed, with a mean (SD) percent of overall work impairment (work time missed [absenteeism] and impairment while working [presenteeism] in the last 7 days) due to asthma of 13.1% (22.4). Overall, 76% of patients had experienced an exacerbation, with 58, 29, and 13% experiencing a mild, moderate, or severe exacerbation in the past 12 months, respectively. Almost all patients agreed with the statement “I believe that it is helpful to categorize asthma attacks or flare-ups by their severity” (97%), and the majority agreed with the statement “I believe that asthma attacks or flare-ups have an impact on my life” (78%). In addition, half of patients (50%) agreed with the statement “I worry about having asthma attacks or flare-ups”.

**Table 1:** Patient demographics and clinical characteristics

Demographics	Patients (*N* = 302)
Age, years, mean (SD)	48.2 (16.7)
Gender, female, *n* (%)	184 (60.9)
Race^a^, *n* (%)	
White	234 (77.5)
Black or African American	30 (9.9)
Other^b^	40 (13.2)
Health insurance^a^, *n* (%)	
Commercial	194 (64.2)
Medicare	77 (25.5)
Medicaid	32 (10.6)
Federal Employee Health Benefits	17 (5.6)
Other^c^	17 (5.6)
Education, *n* (%)	
(Some) high school or equivalent	25 (8.3)
(Some) college	106 (35.1)
Graduate school/degree	169 (56.0)
2016 household income, *n* (%)	
< $50,000	89 (29.5)
$50,000–$99,999	124 (41.1)
≥ $100,000	66 (21.9)
Chose not to answer	23 (7.6)
Clinical characteristics	
Number of years with asthma, *n* (%)	
< 1–10 years	90 (29.8)
11–19 years	56 (18.5)
≥ 20 years	156 (51.7)
Comorbidities^a^, *n* (%)	
Hypertension	95 (31.5)
High cholesterol	91 (30.1)
Anxiety	90 (29.8)
Depression	88 (29.1)
Obesity	78 (25.8)
Type 2 diabetes	31 (10.3)
Cardiovascular disease	8 (2.6)
Current controller medication, *n* (%)	233 (77.2)
Current rescue medication, *n* (%)	239 (79.1)

Data may not add up to 100% due to rounding and the ability of the respondent to select multiple responses

^a^Respondent could select more than one response. ^b^Other includes: Asian, American Indian or Alaskan Native, and Native Hawaiian or Pacific Islander, other race, or chose not to answer. ^c^Other includes: no coverage, unknown or chose not to answer

*SD* standard deviation

**Table 2:** Patient-reported outcomes

Patient-reported outcomes	Patients (*N* = 302)
EQ-5D-5 L domains	
Proportion of patients reporting problems/impact, *n* (%)	
Pain/discomfort	162 (53.6)
Anxiety/depression	142 (47.0)
Usual activities	115 (38.1)
Mobility, ability to walk about	108 (35.8)
Self-care (i.e. washing or dressing)	28 (9.3)
EQ-5D VAS, mean (SD)^a^	74.2 (16.0)
Sleep impact (proportion reporting impact), *n* (%)	149 (49.3)
ACT	
Total ACT score^b^, mean (SD)	20.3 (4.3)
ACT levels of asthma control, *n* (%)	
Poorly controlled (score < 16)	48 (15.9)
Somewhat controlled (score 16–19)	62 (20.5)
Well controlled (score > 19)	192 (63.6)
WPAI	
Currently employed, *n* (%)	171 (56.6)
WPAI summary scores^c^, mean (SD)	
Proportion of respondents missing at least 1 h from work during the past 7 days due to asthma^d^, *n* (%)	16 (9.4)
Percent work time missed due to asthma during the past 7 days (absenteeism)^e^	3.1 (12.7)
Percent impairment while working due to asthma during the past 7 days (presenteeism)^f^	11.8 (20.4)
Percent overall work impairment (absenteeism and presenteeism during the past 7 days)^g^	13.1 (22.4)
Percent impairment, regular daily activities during the past 7 days ^h^	19.3 (24.7)
Patient exacerbations^i^, yes, *n* (%)	
Proportion of respondents who ever experienced an exacerbation, flare-up, or attack	230 (76.2)
A mild exacerbation, flare-up, or attack in the past 12 months	174 (57.6)
Amoderate exacerbation, flare-up, or attack in the past 12 months	89 (29.5)
A severe exacerbation, flare-up, or attack in the past 12 months	39 (12.9)
Patient belief statements, yes, *n* (%)	
I believe it is helpful to categorize asthma attacks or flare-ups by severity (mild, moderate, severe)	292 (96.7)
I believe all patients with asthma are at risk of developing an attack or flare-up	274 (90.7)
I believe asthma attacks or flare-ups can have a varying impact on my life, depending on the severity of the exacerbation	275 (91.1)
I believe asthma attacks or flare-ups have an impact on my life	236 (78.1)
I believe my asthma attacks or flare-ups have an impact on other people in my life	212 (70.2)
I worry about having asthma attacks or flare-ups.	150 (49.7)

^a^Ranges from 0 (worst health imaginable) to 100 (best health imaginable). ^b^Ranges from 5 to 25; higher values indicate better asthma control. ^c^Calculated from patients who indicated they were currently employed, with the exception of “Percent Impairment in Regular Daily Activities in the Past 7 Days”, which was calculated for all respondents. ^d^*N* = 171. ^e^Number of hours missed because of asthma divided by the sum of that number plus the number of hours actually worked. If either value was missing or if both were zero, percent absenteeism was set to missing; *N* = 151. ^f^Presenteeism is only reported for those respondents who entered > 0 h actually worked in the past 7 days; *N* = 150. ^g^*N* = 150. ^h^*N* = 302. ^i^Exacerbation definitions by severity are provided in Additional file 2

*ACT* Asthma Control Test, *EQ-5D-5 L* 5-level quality of life survey instrument developed by EuroQol Group, *EQ-VAS* EQ Visual Analogue Scale, *SD* standard deviation, *WPAI* Work Productivity and Activity Impairment

### HCP characteristics

HCP characteristics are described in Table 3. Over half (56%) were male, 45% had practiced for  > 20 years and the majority were specialized in primary care, general internal medicine, or family practice (80%). In the 12 months prior to the study, a mean of 307 patients with asthma were treated by the HCPs; of these, 52, 34, and 14% had mild, moderate, or severe asthma, respectively. Overall, 75% of HCPs categorized exacerbations by their severity (mild, moderate, severe) and reported treating a mean of 17 patients for an exacerbation in the previous month (50% mild, 37% moderate, and 12% severe exacerbations). Almost all HCPs agreed with the belief statements (≥91%).

**Table 3:** HCP characteristics

Demographic and practice characteristics	HCP (*N* = 300)
Current role, *n* (%)	
Physician (MD or DO)	297 (99.0)
Other^a^	3 (1.0)
Gender, female, *n* (%)	129 (43.0)
Provider specialty^b^, *n* (%)	
Primary care, general internal medicine, family practice	240 (80.0)
Pulmonology/allergy	15 (5.0)
Other^c^	51 (17.0)
Years of practice, *n* (%)	
≤ 10 years	72 (24.0)
11–20 years	92 (30.7)
> 20 years	136 (45.3)
Main practice setting, *n* (%)	
Physician-owned practice	139 (46.3)
Part of a large medical group or healthcare system	80 (26.7)
Hospital or clinic not associated with a university (including community health clinic)	34 (11.3)
University hospital or clinic	29 (9.7)
Group or staff model HMO	10 (3.3)
Other^d^	8 (2.7)
Clinical characteristics and exacerbations^e^	
Categorizes severity of exacerbations as mild, moderate, or severe, yes, *n* (%)	224 (74.7)
Number of patients with asthma treated in the past 12 months, mean (SD)	307.2 (679.8)
Percent of patients with mild asthma	51.5 (21.5)
Percent of patients with moderate asthma	34.4 (15.7)
Percent of patients with severe asthma	14.1 (12.0)
Number of patients with asthma treated for an exacerbation in the past month, mean (SD)	17.5 (40.1)
Percent of patients with mild exacerbations	50.2 (25.4)
Percent of patients with moderate exacerbations	37.5 (21.9)
Percent of patients with severe exacerbations	12.3 (14.0)
Belief statements, yes, *n* (%)	
I believe that asthma exacerbations can have a varying impact on my patients’ lives, depending on the severity of the exacerbation	298 (99.3)
I believe that asthma exacerbations have an impact on my patients’ lives	296 (98.7)
I believe all patients with asthma are at risk of developing an exacerbation	289 (96.3)
I believe that it is helpful to categorize asthma exacerbations by their severity (mild, moderate, severe)	276 (92.0)
I believe my patients worry about having exacerbations	273 (91.0)

^a^Other includes: registered nurse, physician assistant, and acupuncturist. ^b^Respondent could select more than one response. ^c^Other includes: pediatrics, pediatric emergency medicine, pediatric hematology/oncology, pediatric other, urgent care/emergency medicine, obstetrics/gynecology, orthopedics, geriatrics, pathology, radiology and dermatology. ^d^Other includes: direct primary care practice, federally-qualified rural health centers, locum tenens practice, long-term care, physician in private practice, residency clinic associated with a healthcare system, and urgent care. ^e^Exacerbation definitions by severity are provided in Additional file 2

*DO* Doctor of Osteopathic Medicine, *HMO* Health Maintenance Organization, *MD* Doctor of Medicine, *SD* standard deviation

### Vignette outcomes

**Fig. 1:**
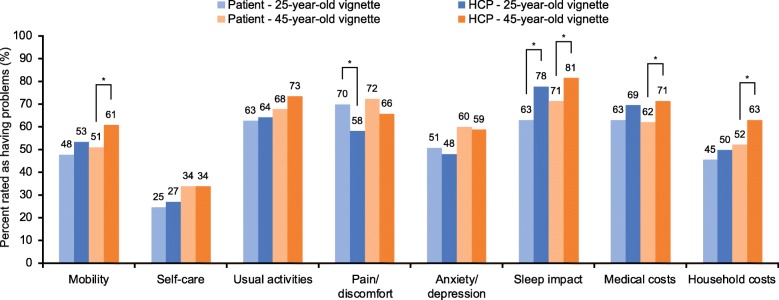
Mild exacerbation vignette impact scores. *p ≤0.05. Problems in each EQ-5D-5 L item is rated as a score of > 1 (on a scale of 1–5). Chi-square tests were used for binary measures, if counts were < 25 then Fisher’s Exact chi-square test was used

**Fig. 2:**
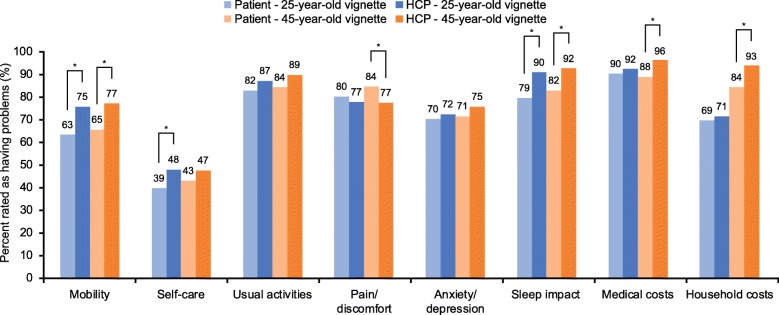
Moderate exacerbation vignette impact scores. *p ≤0.05. Problems in each EQ-5D-5 L item is rated as a score of > 1 (on a scale of 1–5). Chi-square tests were used for binary measures, if counts were < 25 then Fisher’s Exact chi-square test was used

**Fig. 3:**
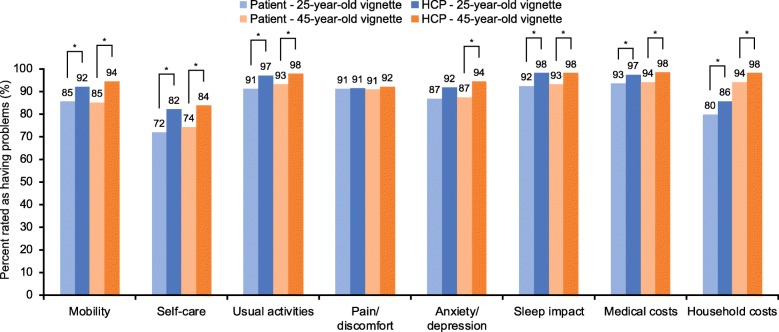
Severe exacerbation vignette impact scores. *p ≤0.05. Problems in each EQ-5D-5 L item is rated as a score of > 1 (on a scale of 1–5). Chi-square tests were used for binary measures, if counts were < 25 then Fisher’s Exact chi-square test was used

The proportion of patients reporting impact for the eight burden domains (EQ-5D-5 L domains, sleep impact and financial burden) increased with increasing severity of exacerbations. Significantly more patients than HCPs indicated problems with pain/discomfort for the vignettes of the 25-year-old patient experiencing a mild exacerbation (70% vs 58%, *p* = 0.003; Fig. 1) and for the vignettes of the 45-year-old patient experiencing a moderate exacerbation (84% vs 77%, *p* = 0.031; Fig. 2). Significantly more HCPs than patients indicated that exacerbations impacted sleep for both the vignettes of the 25-year-old and 45-year-old patients experiencing mild (25-year-old: 78% vs 63%, *p* < 0.001; 45-year-old: 81% vs 71%, *p* = 0.004) or moderate exacerbations (25-year-old: 90% vs 79%, *p* < 0.001; 45-year-old: 92% vs 82%, *p* < 0.001) (Figs. 1 and 2). Compared with patients, a greater proportion of HCPs indicated an impact on mobility and economic burden (medical and household) for the vignettes of the 45-year-old patient experiencing mild or moderate exacerbations, as well as issues with mobility and self-care for the vignettes of the 25-year-old patient experiencing a moderate exacerbation (Figs. 1 and 2). For both severe exacerbation vignettes, HCPs were more likely to report impact on mobility, self-care, usual activities, sleep, and medical costs compared with patients (Fig. 3). Issues with anxiety/depression and household costs were also more likely to be identified by HCPs in the vignettes of the 45-year-old patient compared with patients.

## Discussion

This study examined the perceived impact of asthma exacerbations from the perspectives of a US sample of patients with asthma and HCPs who treat patients with asthma. In our study, despite the lack of agreed standards for defining exacerbations or their severity, there were some commonly held views among patients and HCPs. Both agreed that there is an increasing impact as exacerbation severity increases, with severe exacerbations having a greater impact on all aspects of a patient with asthma life than mild or moderate exacerbations. The greatest perceived impact of mild exacerbations was pain/discomfort for patients and sleep for HCPs. The greatest perceived impact of moderate exacerbations was medical costs for both patients and HCPs, whereas severe exacerbations were perceived as having the greatest impact on medical costs by patients and on sleep, medical costs, and usual activities by HCPs. There was also a strong consensus among patients and HCPs that it is helpful to categorize asthma exacerbations by their severity, and a strongly shared belief that asthma attacks can have varying impact on patients’ lives, depending on the severity of the exacerbation.

Shared decision-making between patients and HCPs is recommended by the Global Initiative for Asthma (GINA 2018) treatment guidelines to improve symptom control; however, differences in perspectives between patients and HCPs are common and may contribute to poorer outcomes in patients with asthma [3, 20]. In this study, several key differences in perceived impact of the EQ-5D-5 L, sleep and financial burden measures were identified between patients with asthma and HCPs, with HCPs tending to perceive exacerbations as more burdensome compared with the patients’ perceptions. Recent studies outside the US have also demonstrated that discordance exists between patients with asthma and HCPs regarding perceptions of control, and the impact of asthma on the daily lives of patients [20–22]. In this study, HCPs were overall aware of the impact of exacerbations on patients beyond the impact on lung function; however, patients were more likely to indicate problems with pain/discomfort compared with HCPs, particularly for mild and moderate exacerbations. This is consistent with previous findings which demonstrated that pain/discomfort was the most common patient-reported problem on the EQ-5D [23], suggesting that, despite the array of published studies on the impact of asthma on patients’ HRQoL, many HCPs remain unaware that patients experience considerable pain and discomfort related to asthma. These findings highlight a need for increased awareness and education of HCPs on the impact of asthma exacerbations, particularly in terms of patient perceived pain and discomfort.

Potential reasons for the differences observed between patient and HCP perceptions may include miscommunication, patients downplaying symptoms, or patients not reporting all exacerbations (particularly mild or moderate) to their HCPs. As such, patients may have a more holistic view of the burden attributed to exacerbations, whereas HCPs are likely only aware of the events for which patients have sought treatment. Both groups agree on the substantial impact of severe exacerbations; however, this study has highlighted that it is important for patients who are experiencing mild, and particularly moderate, exacerbations to see their physicians so that exacerbation episodes can be effectively managed before they become more severe and so the impact of their current exacerbation and risk of future exacerbations are minimized.

One factor which may contribute to the gaps in communication between patients and HCPs is the lack of clearly defined levels of severity for asthma-related exacerbations in clinical practice. Exacerbations are currently defined based on healthcare utilization and not patient experience, and the lack of ability to link healthcare utilization to patient experience presents a specific challenge. This study used its own definition for asthma-related exacerbations, operationalizing a picture of what a mild, moderate, or severe exacerbation looked like using healthcare use as the marker of severity. Both patients and HCPs agreed that categorizing exacerbations by severity was helpful as they can have varying impacts (health-related and economic) on the patients’ lives according to severity. This highlights a need for a clear definition of asthma-related exacerbations rooted in patient experience in clinical practice and which considers patient-reported experiences such as HRQoL and impact on work.

Limitations of this study included those typically associated with survey-based studies. The study relied upon self-reported diagnoses of asthma, which were not confirmed by medical record review. In addition, participants were members of opt-in online panels who participated in survey research, so may not be fully representative of all patients with asthma or all HCPs who treat patients with asthma. The patient sample in this study appears to be more representative of patients with mild asthma and was more closely matched in terms of age to Patient B than Patient A; these factors may have impacted the vignette responses. Similarly, 24% of responding patients reported never having experienced an exacerbation; however, 91% of patients who completed the vignette exercise also indicated that they were familiar with the term and need to categorize exacerbations by severity (97%) and were therefore likely to understand the potential impact of these exacerbations. Furthermore, the population of HCPs who participated were largely family/primary care practitioners and therefore may not be representative of asthma care by respiratory specialists. Finally, this study did not adjust for multiplicity and multiple comparisons could increase the likelihood of statistically significant results; however, as this was a descriptive, hypothesis-generating study, this was not considered necessary for the intended analysis.

## Conclusion

Asthma-related exacerbations can have a significant impact on patients’ lives; however, the perceived impact of these exacerbations differs between patients and HCPs and this may contribute to suboptimal clinical outcomes. Across all exacerbation severity levels, HCPs perceived a higher degree of impact than patients; however, they may be less aware of their patients’ concerns regarding pain and discomfort when experiencing mild or moderate exacerbations. This study highlights a need for a clear definition for asthma-related exacerbations in clinical practice that considers patient-reported experiences. Further studies are needed to understand differences in patient and HCP perspectives and patient-HCP interactions regarding asthma-related exacerbations, which may be important considerations for optimizing patient care.

## Additional files

Additional file 1: Vignette case descriptions. List of case descriptions. (DOCX 31 kb)

Additional file 2: Exacerbation definitions. Table of exacerbation definitions. (DOCX 18 kb)

## Data Availability

This study was a collaboration between GSK and Optum. GSK makes available anonymized individual participant data and associated documents from interventional clinical studies which evaluate medicines, upon approval of proposals submitted to www.clinicalstudydatarequest.com. To access data for other types of GSK-sponsored research, for study documents without patient-level data and for clinical studies not listed, please submit an enquiry via the website. For this manuscript, the data is contained in a database owned by Optum and contains proprietary elements and, therefore, cannot be broadly disclosed or made publicly available at this time. The disclosure of this data to third-party clients assumes certain data security and privacy protocols are in place and that the third-party client has executed Optum’s standard license agreement which includes restrictive covenants governing the use of the data.

## Abbreviations

ACT: Asthma Control Test
ATS/ERS: American Thoracic Society/European Respiratory Society
CASIS: COPD and Asthma Sleep Impact Scale
DO: Doctor of Osteopathic Medicine
EQ-5D-5 L: 5-level QoL survey instrument developed by the EuroQol Group
EQ-VAS: EQ-Visual Analogue Scale
GINA: Global Initiative for Asthma
HCP: Healthcare provider
HMO: Health Maintenance Organization
HRQoL: Health-related quality of life
IRB: Institutional Review Board
MD: Doctor of Medicine
PROs: Patient-reported outcomes
SD: Standard deviation
US: United States
WPAI: Work Productivity and Activity Impairment
