# Supplementary material for: Observational vignette study to examine patient and healthcare provider perceived impact of asthma-related exacerbations in the US
Source: Multidiscip Respir Med. 2019 Nov 5;14:32. doi: 10.1186/s40248-019-0196-1 (PMC6829825; doi:10.1186/s40248-019-0196-1)
Supplement: Supplementary file 1 — Vignette case descriptions. List of case descriptions. (DOCX 31 kb) [file 40248_2019_196_MOESM1_ESM.docx]

**Additional File 1: Vignette case descriptions**

**Case description #1:** Patient A is a 25-year-old college student who is single, not employed, and has no dependents. Patient A had an asthma flare-up today that they treated on their own by increasing their use of rescue medications and inhaled corticosteroids.

**Case description #2:** Patient B is a 45-year-old, married parent of two young children who also works full time. Patient B had an asthma flare-up today that they treated on their own by increasing their use of rescue medications and inhaled corticosteroids.

**Case description #3:** Patient A is a 25-year-old college student who is single, not employed, and has no dependents. Patient A had a worsening of asthma symptoms that required an office visit with their doctor today. Patient A’s doctor prescribed extra controller medications and an additional steroid medication (oral or injected).

**Case description #4:** Patient B is a 45-year-old, married parent of two young children who also works full time. Patient B had a worsening of asthma symptoms that required an office visit with their doctor today. Patient B’s doctor prescribed extra controller medications and an additional steroid medication (oral or injected).

**Case description #5:** Patient A is a 25-year-old college student who is single, not employed, and has no dependents. Patient A had a worsening of asthma symptoms that required going to the hospital where they are now being treated with intravenous steroids or prednisone, prednisolone, or another corticosteroid. Patient A will need to be treated with the steroid medications for at least
5–7 days.

**Case description #6:** Patient B is a 45-year-old, married parent of two young children who also works full time. Patient B had a worsening of asthma symptoms that required going to the hospital where they are now being treated with intravenous steroids or prednisone, prednisolone, or another corticosteroid. Patient B will need to be treated with the steroid medications for at least 5–7 days.
