# Supplementary material for: Observational vignette study to examine patient and healthcare provider perceived impact of asthma-related exacerbations in the US
Source: Multidiscip Respir Med. 2019 Nov 5;14:32. doi: 10.1186/s40248-019-0196-1 (PMC6829825; doi:10.1186/s40248-019-0196-1)
Supplement: Supplementary file 2 — Exacerbation definitions. Table of exacerbation definitions. (DOCX 18 kb) [file 40248_2019_196_MOESM2_ESM.docx]

**Additional File 2: Exacerbation definitions**

HCP, healthcare provider; IV, intravenous

| **Exacerbation severity** | **Definition**  ***A worsening of asthma***  ***symptoms that…*** | **Patient A (25 year old) and**  **Patient B (45 year old) vignettes** |
| --- | --- | --- |
| **Mild** | …may not require seeing a HCP and can usually be managed by increased use of rescue medications (and sometimes the start or the increase in inhaled steroid medication dosage). | “Patient A/B had a flare-up today that they **treated on their own** by **increasing** their use of **rescue medications** and **inhaled corticosteroids.**” |
| **Moderate** | …requires seeing a HCP and typically treated with rescue medications on top of extra controller medications and/or a systemic steroid (oral or injection) for a short period of time along with the possible need for an antibiotic. | “Patient A/B had a worsening of asthma symptoms that required an **office visit with their doctor** today. The doctor prescribed Patient A/B to take **extra controller medications** and an **additional steroid medication** (oral or injected).” |
| **Severe** | …requires going to an emergency room and/or being hospitalized and treated with a systemic steroid via intravenous or oral forms (prednisone, prednisolone, or another corticosteroid) for usually more than 5–7 days along with or without an antibiotic. | “Patient A/B had a worsening of asthma symptoms that required **going to the hospital** where they are now being treated with **IV steroids or prednisone, prednisolone, or another corticosteroid**. Patient A/B will need to be treated with the **steroid medications for at least 5–7 days**.” |
